# Dr. Hunter Pope Cooper

**Published:** 1907-02

**Authors:** E. C. Davis, W. S. Goldsmith


### DR. HUNTER POPE COOPER.

---

To have lived a natural life and to be taken away when one's life task has been completed leaves its sorrow and griefs more easily borne than when one in the very acme of usefulness is taken away when friends and family seem so much to need them. Such was the case with Dr. Cooper when he was bordering upon the zenith of usefulness in his profession and life he was taken away, illustrating the inexplicable plan of the Creator. He had in reality just entered upon that period of his professional life when he was the most capable of doing the most good to suffering humanity. The life of a student, ripe with broad experience and methodical in his plans, cannot fail to accomplish valuable results; add to this a very positive social nature and an intensive purpose, and we have some of the characteristics of Dr. Cooper.

No man has been taken away from the local profession who will be missed more, for he was a man whom you could always tell where he stood on any question, as he was positive and firm in his convictions, probably the most attractive lecturer and magnetic leader in the South; his students were fond of quoting him and his lectures were gems of thought and logically arranged. As a practitioner, he was a signal success, having one of the mose select and lucrative practices of any one in this section. As a friend, he endeared himself most closely to those with whom he was thrown; he was ever honest, faithful and cultured, and those of his clientele adhered most closely to him throughout his professional life.

A little sketch of his life would not be out of place, and is as follows:

Hunter Pope Cooper, born in Atlanta, May 16th, 1960; son of Thomas L. Cooper, Solicitor-General, Atlanta Circuit; died August 24th, 1906. Preparatory education under Prof. C. M. Neel, Kirkwood High School. He later attended the University of Georgia and the University of Virginia. Medical education received at the College of Physicians and Surgeons, New York, and served as interne in the Presbyterian Hospital, New York. Later, post-graduate work in Vienna and Berlin. In 1886 located in Atlanta, and in 1888 was elected to the Chair of Chemistry in the Atlanta Medical College. He succeeded the late Dr. Armstrong in the Chair of Anatomy in the same institution, and later was elected to succeed the late Dr. Hardon in the chair of Obstetrics and Gynecology in the Atlanta College of Physicians and Surgeons. For twelve years he was surgeon at the Grady Hospital, and was also gynecologist to the Wesley Memorial Hospital In 1897, he became associated with Dr. W. S. Elkin, when they established a private sanatorium.

He married Miss Henrietta Tucker, daughter of the late Rev. Henry H. Tucker. He leaves a widow, one daughter, Mary, and one son, Thomas L. Cooper, Jr.

He was a member of the Fulton County Medical Society, Georgia State Medical Association and the American Medical Association. He was Chief Surgeon of the Atlanta & West Point Railroad and Western Railway of Alabama, Division Surgeon of the Nashville, Chattanooga & St. Louis Railway, and Local Surgeon of the Georgia and the Louisville & Nashville Railroads.

In the loss of such a man, this Society has lost one of its most valued members, the profession has sustained an almost irreparable loss, and the home and family have sustained a grief beyond repair. We, therefore, request that this brief sketch be put upon the records of this Society, a copy sent to the bereaved family, and a copy sent to the Atlanta Journal-Record of Medicine, for publication.

E. C. DAVIS,
W. S. GOLDSMITH.

---

Do not operate for foreign body in the knee joint without first excluding dislocation of one of the semilunar cartilages.—*American Journal of Surgery.*

---

In amputation below the knee, insist on active and passive motion in the knee joint at an early date. If this is not done contracture ensues, which makes the application of an artificial limb difficult.—*American Journal of Surgery.*

---

In compound fractures involving loss of continuity do not needlessly remove any piece of bone that has even the smallest attachment. It is surprising how often such pieces heal into the wound and thereby help to save loss of substance.—*American Journal of Surgery.*

---

Fractures of the neck of the femur in old people sometimes cause no other syptoms than disability. The mildness of the trauma and the freedom from much pain should not deceive one.—*American Journa of Surgery.*

---

The radiograph of the elbow of a child shows shadows of numerous epiphysis. One inexperienced with the x-ray plates is very apt to mistake one or more of these for fractures. When examining the skiagraph of a child's elbow suspected of fracture or dislocation, it is, therefore, important to have the normal picture in mind, or better yet in hand, for comparison.—*American Journal of Surgery.*

